# Changes in CRH and ACTH Synthesis during Experimental and Human Septic Shock

**DOI:** 10.1371/journal.pone.0025905

**Published:** 2011-11-03

**Authors:** Andrea Polito, Romain Sonneville, Céline Guidoux, Lucinda Barrett, Odile Viltart, Virginie Mattot, Shidasp Siami, Geoffroy Lorin de la Grandmaison, Fabrice Chrétien, Mervyn Singer, Françoise Gray, Djillali Annane, Jean-Philippe Brouland, Tarek Sharshar

**Affiliations:** 1 Department of Intensive Care, Raymond Poincaré Hospital, Garches, France; 2 Laboratory of Neuroendocrin Response to Sepsis, EA4342, University Versailles Saint-Quentin en Yvelines, Garches, France; 3 Department of Pathology, Lariboisière Hospital, Paris, France; 4 Department of Infection and Epidemiology, HISTO (Human hISTOpathology and animal models), Pasteur Institute, Paris, France; 5 Department of Intensive Care, University College, London, United Kingdom; 6 Department of Biology, CNRS-UMR8161, Pasteur Institute of Lille, Lille, France; 7 Department of Plasticity of the Postnatal Brain, INSERM U837, University of Nord de France, Lille, France; 8 Department of Pathology, Raymond Poincaré Hospital, Garches, France; 9 Departement of Medicine, University College, London, United Kingdom; Rutgers University, United States of America

## Abstract

**Context:**

The mechanisms of septic shock-associated adrenal insufficiency remain unclear. This study aimed at investigating the synthesis of corticotropin-releasing hormone (CRH) and vasopressin (AVP) by parvocellular neurons and the antehypophyseal expression of ACTH in human septic shock and in an experimental model of sepsis.

**Objective:**

To test the hypothesis that ACTH secretion is decreased secondarily to alteration of CRH or AVP synthesis, we undertook a neuropathological study of the antehypophyseal system in patients who had died from septic shock and rats with experimental faecal peritonitis.

**Methods:**

Brains obtained in 9 septic shock patients were compared to 10 nonseptic patients (controls). Parvocellular expression of AVP and CRH mRNA were evaluated by in situ hybridization. Antehypophyseal expression of ACTH, vasopressin V1b and CRH R1 receptors and parvocellular expression of iNOS in the PVN were evaluated by immunohistochemistry. The same experiments were carried out in a fecal peritonitis-induced model of sepsis. Data from septic rats with (n = 6) or without (n = 10) early death were compared to sham-operated (n = 8) animals.

**Results:**

In patients and rats, septic shock was associated with a decreased expression of ACTH, unchanged expression of V1B receptor, CRHR1 and AVP mRNA, and increased expression of parvocellular iNOS compared to controls. Septic shock was also characterized by an increased expression of CRH mRNA in rats but not in patients, who notably had a greater duration of septic shock.

**Conclusion:**

The present study suggests that in humans and in rats, septic shock is associated with decreased ACTH synthesis that is not compensated by its two natural secretagogues, AVP and CRH. One underlying mechanism might be increased expression of iNOS in hypothalamic parvocellular neurons.

## Introduction

About sixty percent of patients with septic shock develop adrenal insufficiency [1] and subsequently may have higher risk of death [2], [3]. The mechanisms of impairement of the hypothalamo-pituitary-adrenal axis (HPA) during critical illness are complex and poorly understood and may include decreased production of corticotropin-releasing hormone (CRH), adreno-corticotropic hormone (ACTH) and cortisol as well as dysfunction of their respective receptors [4]. The antehypophyseal system includes the parvocellular neurons of the hypothalamic paraventricular nuclei (PVN) producing CRH and vasopressin (AVP), and the antehypophysis where ACTH is synthesised. CRH neurons in the PVN project to median eminence, where CRH is released into the hypophyseal portal circulation to stimulate ACTH secretion via essentially CRH receptor 1 (CRHR1), which is the most abundant form of CRH receptors [5], [6]. AVP produced by the parvocellular neurons also controls ACTH secretion via V1b receptor [7].

Most human studies of HPA axis during sepsis have relied on circulating hormonal levels and have never included neuropathological studies. Previous experimental studies investigated hypothalamo-pituitary structures and showed that endotoxin or cytokines stimulate CRH neurons [8]. However, they rarely assessed concomitantly the expression of AVP and CRH and their respective receptors as well as relationships between HPA activation and the severity of sepsis.

In order to test the hypothesis that ACTH secretion is decreased secondarily to alteration of either CRH or AVP synthesis, we underwent a neuropathological study of the antehypophyseal system in patients who had died from septic shock and rats with experimental faecal peritonitis, which is considered one of the best model of human sepsis [9]. Faecal peritonitis enables to obtain various degree of sepsis severity [10], [11] and seems an appropriate model for assessing pathophysiological mechanisms of sepsis-induced hormonal disturbances [12], [13].

## Methods

### In Vivo Model of Fecal Peritonitis

Experiments were carried out at University College London, with Home Office approval in accordance with the Animal Scientific Procedures Act (European Community's Council Directive (24/11/1986; 86/609/EEC). Wistar rats were purchased from Charles River laboratory (Arbresle, France). This model has been well described elsewhere [11]. Briefly, anesthetised male Wistar rats were instrumented to develop sepsis by intraperitoneal injection of fecal slurry (0.63 mg/100 g body weight) prepared from the bowel contents of a rat from the same batch. Fluid resuscitated rats were randomly divided as follows: 1) sham-operated controls that received no intraperitoneal injection but were otherwise treated identically; 2) Rats that underwent experimental peritonitis and were classified as severe sepsis if they did not die spontaneously (i.e. septic rats) and as septic shock if they died spontaneously (i.e. septic early death (ED) rats). Sham and septic rats were killed by cervical dislocation at 24 or 48 hours after injection of slurry. All septic shock rats died within 36 hours. All septic and septic ED animals showed features of illness from about 12 h after injection of fecal slurry, including hunched posture, piloerection, and decreased movement and alertness. At 24 hours, there was a significant decrease in mean arterial pressure in septic rats (115±2 mmHg controls, 106±4 mmHg septic, p<0.05). In all septic and septic shock rats, laparotomy showed evidence of peritoneal inflammation.

Brain sampling was performed in eight sham, ten septic and six septic shock rats. Whole brains and pituitary glands were removed immediately after animal sacrifice by neck dislocation, and fixed by immersion in 10% formalin. Brain samples were processed as previously described [14]. Briefly, after embedding in paraffin, serial sections of 4 µm were performed (Leica® microtome) until the region of interest was reached. Coronal sections of the PVN nuclei and horizontal sections of the pituitary gland were then stained with haematoxylin and eosin before microscopic examination or treated for immunohistochemistry or *in situ* hybridization.

### Patients

Nine non-survivors from septic shock and ten non-septic patients who died from cardiogenic shock or an extracranial cause of sudden death were included. The characteristics of these patients have been reported elsewhere (10). The median age of patients who died from septic shock was 71 [58–76] years. Their SAPS-II score was 57 [35–90], the cumulative time of hypotension was 25 [12–36] h and duration of shock 6 [1–8] days. In patients who died from non-septic causes, SAPS-II score was higher (i.e. 70 [47–83]) but cumulative time of hypotension and duration of shock were shorter, namely 3 [2–5] h and 3 [1–4] days, respectively.

Macroscopic examination of the brains was performed after 4 to 6 weeks of fixation in 10% formalin (vol./vol.). We took samples of the PVN at the level of the posterior hypothalamus. Pituitary glands were also sampled. Each sample was embedded in paraffin and cut in 4 µm serial sections with a Leica® microtome. For microscopic examination, one section on three was stained with haematoxylin and eosin; the others were processed for immunohistochemistry or *in situ* hybridization.

### Human ethics

According to national regulations, brain samples were collected after obtaining informed written consent from patient's closest relatives. A protocol about sampling for research purpose by title *Anatomopathological study of neuroendocrine and neurovegetative trouble during septic shock (n°PFS10-008)* was approved by the French Biomedecine Agency.

Moreover, patients of our work from Raymond Poincaré Hospital (Garches) were enrolled in the CORTICUS study [15]. Written informed consent was provided from a surrogate, the next of kin, or a legal representative (with retrospective consent obtained from patients who regained competency) if lacked mental competency. CORTICUS provided an anatomopathological study and was approved by the Comité Consultatif de Protection des Personnes se Prêtant à la Recherche Biomédicale de Saint Germain en Laye, France.

### Immunohistochemistry (IHC)

Immunohistochemistry was performed on deparaffinised formalin-fixed sections using an indirect ABC-peroxidase method revealed with 3-3′diaminobenzidine (DAB) or amino-ethyl-carbazol (AEC) as chromogenes [14]. For homogeneity of labelling, immunohistochemistry was performed using an automated IHC slide-staining system (NexES® IHC Full system, Ventana Medical Systems, Tucson, AZ, USA). Primary antibodies and species specificities are summarized in table 1. Sections were incubated for 30 min with primary antibodies. Slides were then counterstained with bluing reagent for 4 minutes and mounted in Eukitt® for microscopic evaluation. Omission of the primary antibodies and replacement by isotype-matched irrelevant antibody was used for negative control.

**Table 1:** Antibodies.

Antibody	Manufacturer	Reference	Specificity*
AVP	Chemicon® international, CA, USA	AB1565	H, R
ACTH	Chemicon® international, CA, USA	CBL56	H, R
iNOS	USBiological, MA, USA	I7570-31	H, R
V1b R	ALPHA DIAGNOSTIC, TX, USA	AVP1B13-5	H, R
CRHR1	Acris antibodies GmbH HERFORD, GERMANY	SP4643P	H, R

*H: human; R: rat.

Images were acquired using a Leitz Diaplan® microscope (Solms, Germany) equipped with a Nikon Coolpix 950® digital camera (Champigny sur Marne, France).

### *In situ* hybridization

*In situ* hybridization was performed using a 200 bp rat vasopressin cDNA probe (gift from Dr C Rabadan-Diehl, Bethesda, MD, USA) and 1 kb rat CRH cDNA probe (gift from Dr Mayo, Evanston, IL, USA) as described previously [14]. Briefly, sense and antisense probes were synthesized from linearized plasmids using digoxigenin-UTP (Roche, France) and riboprobe combination system (Promega, France). *In situ* hybridization was performed using the Ventana Discovery® (Ventana Medical Systems, Tucson, AZ, USA). Slides were finally mounted with Vectashield mounting medium (Vector Labs-AbCys, Paris, France). The labelled cells were observed with an Axioplan 2 microscope (Zeiss, Göttingen, Germany) equipped with a video camera (Micromax 1MHz, Kodak chip). Images were acquired with AxioVision acquisition and analysis software. Sections hybridized with sense probes showed no staining.

### Evaluation of immunostaining and *in situ* hybridization

Immunostaining and *in situ* hybridization were assessed by two independent observers (AP, RS), blind to rats and patients' groups. Counting of parvocellular neurones was performed morphometrically using Image J 1.36b® software (Wayne Rasband, National Institute of Health, Bethesda, MD). For rat analyses, cell counts were performed on one 4 µm-treated slice containing the PVN (at the level −1.4 or −1.8 mm from Bregma, according to the stereotaxic rat brain atlas of Paxinos and Watson, 1997 [16]). For humans, one slice representative of PVN was selected for counting.

**Figure 1:**
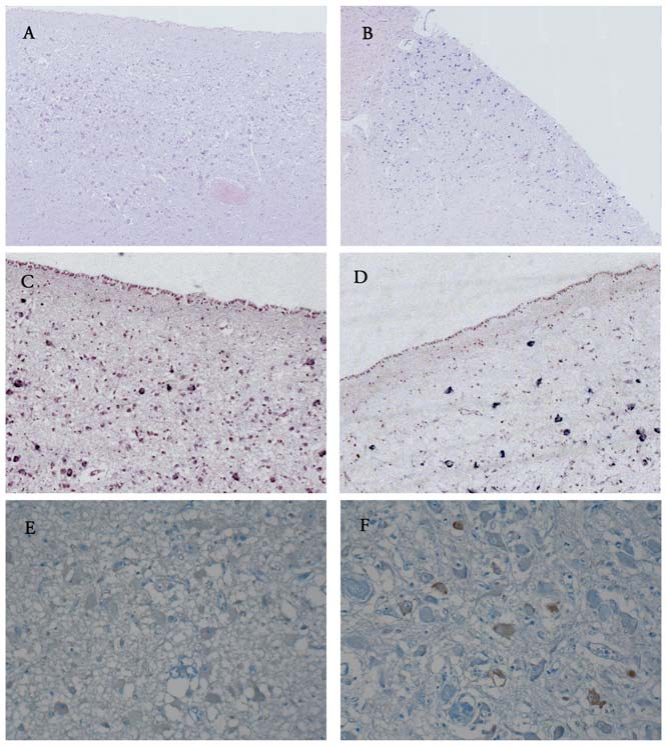
PVN of patients who died from non-septic causes (A–C–E) or septic shock (B–D–F). CRH mRNA (A–B) and AVP mRNA (C–D) labelling after *in situ* hybridization expression did not differ between the two groups. iNOS (E–F) expression after immunohistochemistry (ABC peroxidase/DAB) was higher in septic shock patients.

The intensity of CRH (*in-situ* hybridization), AVP (*in-situ* hybridization) and iNOS (immunohistochemistry) labelling of each neuron was semi-quantitatively scored as 0 (none), 1 (low), 2 (moderate) or 3 (high) in the PVN (Fig. 1 A, B). To take into account both the amount of positive neurones and the cellular intensity of immunostaining, the following index was then calculated for each nucleus: (1*number (n) of low intensity cells + 2*n of moderate intensity cells +3*n of high intensity cells)/(total n of positive and negative neurons) [14], [17]. Therefore, this index ranged from 0 to 3. This index was developed using the hypothalamic nuclei of naïve rats and from patients who did not die from septic shock (Figure 1).

In the antehypophysis of each rat or patient, stores of ACTH, AVP, V1B receptor and CRH1 receptor were scored with a semi-quantitative scale, ranging from 0 to 3 (pourquoi cette échelle ne va pas de 0 à 3 ??), depending on the global immunostaining intensity at the level of the section considered.

### Statistical analysis

Variables were expressed as median with interquartile ranges (25^th^–75^th^ percentile), respectively. Non parametric analyses were performed, using Kruskall-Wallis and Mann-Whitney test for comparison of quantitative variables between three and two groups, respectively. To account for multiplicity while testing the same physiological hypothesis in humans and rats, p-vlaues were adjusted using Holm's correction. A *p*-value less than 0.05 was deemed significant.

## Results

### Expression of CRH, AVP and iNOS in the parvocellular nuclei

In patients, parvocellular expression of CRH mRNA did not differ statistically between septic shock and controls (*p* = 0.65) (Table 2 and Figure 1).

**Table 2:** Parvocellular expression of AVPmRNA, CRHmRNA and iNOS in control and septic shock patients and in sham, septic and septic ED rats.

	Humans			Rats			
	Controls	Septic Shock	p	Sham	Septic	Septic ED	p
CRH mRNA	1.8[1.5–2.0] Fig. 1A	1.8[1.7–1.8] Fig. 1B	0.65	1.6[1.6–1.7] Fig. 2A	1.8[1.6–1.8] Fig. 2B	b2.0[1.9–2.0] Fig. 2C	0.04
AVP mRNA	1.3[1.3–1.4] Fig. 1C	1.4[1.3–1.5] Fig. 1D	1.00	2.1[2.0–2.3] Fig. 2D	2.3[2.0–2.3] Fig. 2E	2.1[1.9–2.3] Fig. 2F	1.00
iNOS	0.0[0.0–0.0] Fig. 1E	1.0[1.0–1.0] Fig. 1F	0.0008	0.0[0.0–2.0] Fig. 2G	a1.0[1. –1.3] Fig. 2H	b3.0[2.3–3.0] Fig. 2I	0.007

Abbreviations: AVP, Vasopressin; CRH, Corticotropin Releasing Hormone; mRNA, messenger RNA; iNOS, inducible nitric oxide synthase; ED, Early death.

Results are expressed as median (IQR).The index of labelling CRH and AVP *in-situ* hybridization and iNOS immunohistochemistry ranged from 0 to 3.

Comparison between the following animal groups:

a Sham *versus* Sepsis,

b Sepsis *versus* Septic Shock,

c Sham *versus* Septic Shock.

**Figure 2:**
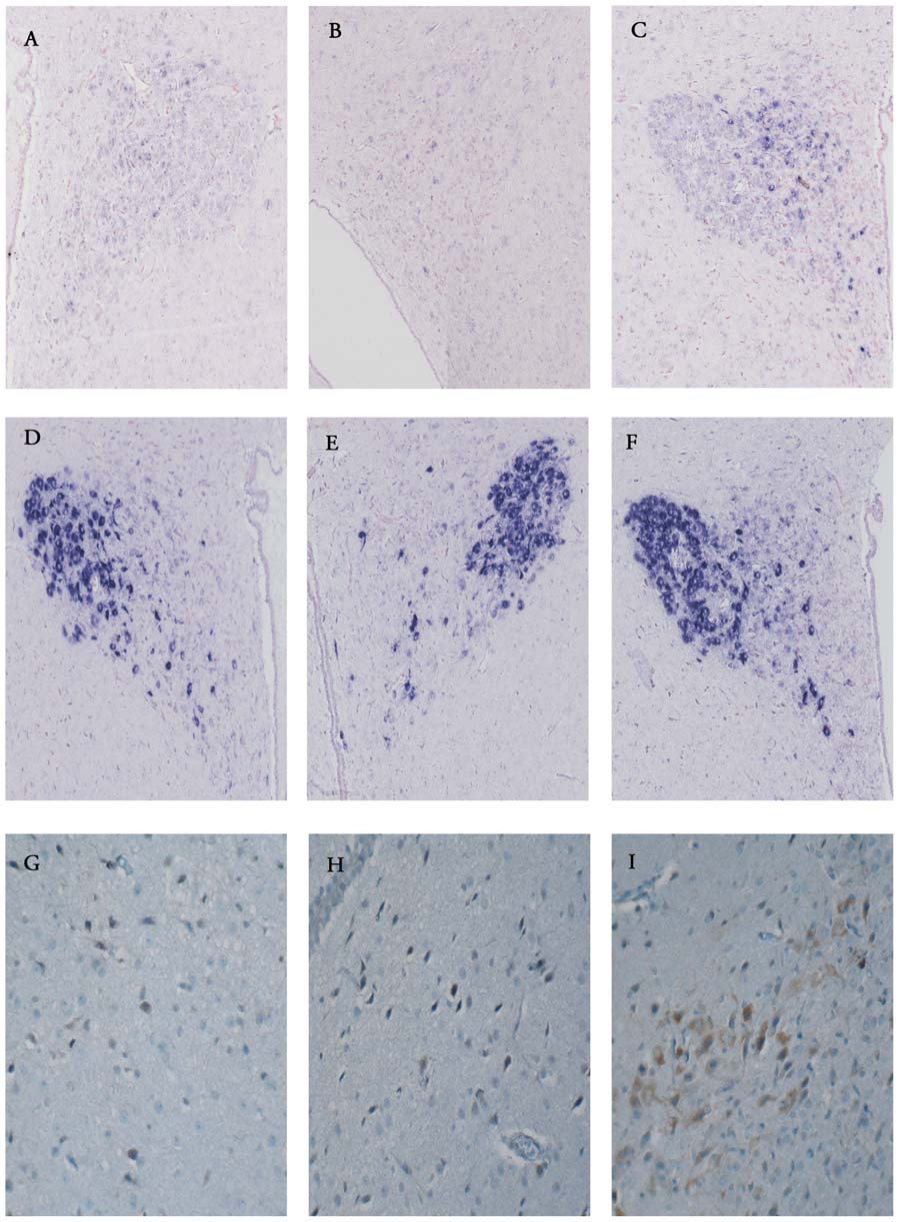
PVN of sham (A–D–G), septic (B–E–H) and septic with early death (septic ED) (C–F–I) rats. Expression of CRH mRNA (A–B–C) and iNOS (G–H–I) after immunohistochemistry (ABC peroxidase/DAB) was greater in septic ED rats. AVP mRNA (D–E–F) expression after *in situ* hybridization did not differ between the three groups.

By contrast, in rats the parvocellular expression of CRH mRNA differed significantly between groups (*p* = 0.04), and was higher in septic shock than in sham (*p* = 0.02) and tended to be higher than septic animals (*p* = 0.08) (Table 2 and Figure 2). It did not differ between sham and septic rats (*p* = 0.21).

In patients, parvocellular expression of AVP mRNA did not differ statistically between septic shock and controls (*p* = 1.00) (Table 2 and Figure 1). Similarly, in rats parvocellular expression of AVP mRNA did not differ between septic and non-septic animals (*p* = 1.00) (Table 2 and Figure 2).

In patients, the index of parvocellular neurons immunoreactive for iNOS was significantly higher in septic shock than in controls (*p* = 0.0008) (Table 2 and Figure 1). Similarly, in rats, the index of parvocellular neurons immunoreactive for iNOS differed significantly between groups (*p* = 0.007) (Table 2 and Figure 2). It was significantly higher in septic shock than in septic (*p* = 0.01) animals and sham (*p* = 0.01) animals. It was not significantly different between septic and sham rats (*p* = 0.43).

In parvocellular nuclei expression of CRH mRNA, AVP mRNA and iNOS in patients did not correlate with duration of septic shock. Evaluation of steroid effect was not possible because of the small number of patients treated.

### Expression of CRHR1, V1b and ACTH in the ante-hypophysis

**Figure 3:**
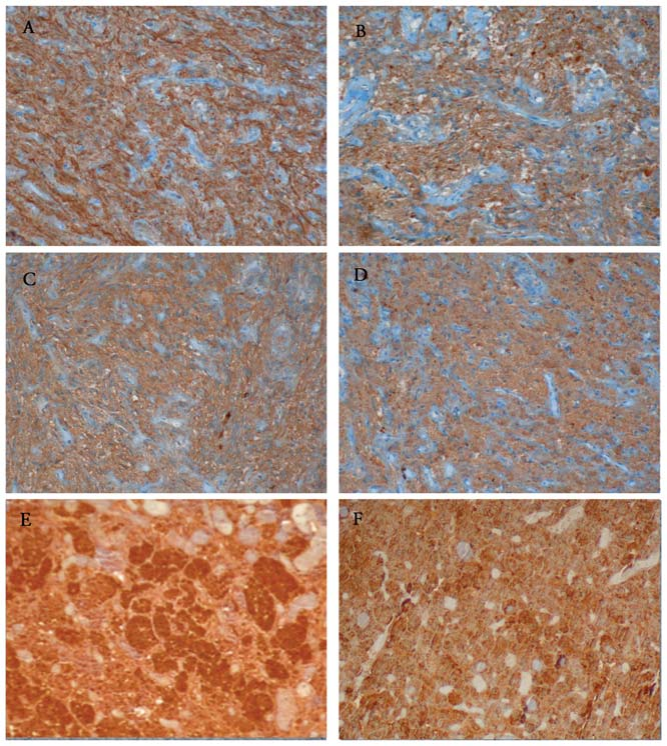
Ante-hypophysis of patients who died from non-septic causes (A–C–E) or septic shock (B–D–F). Labelling of CRHR1 (A–B) and V1b receptor (C–D) after immunohistochemistry (ABC peroxidase/DAB) did not vary among the two groups. ACTH (E–F) immunostaining (ABC peroxidase/DAB) was decreased in septic shock patients.

**Figure 4:**
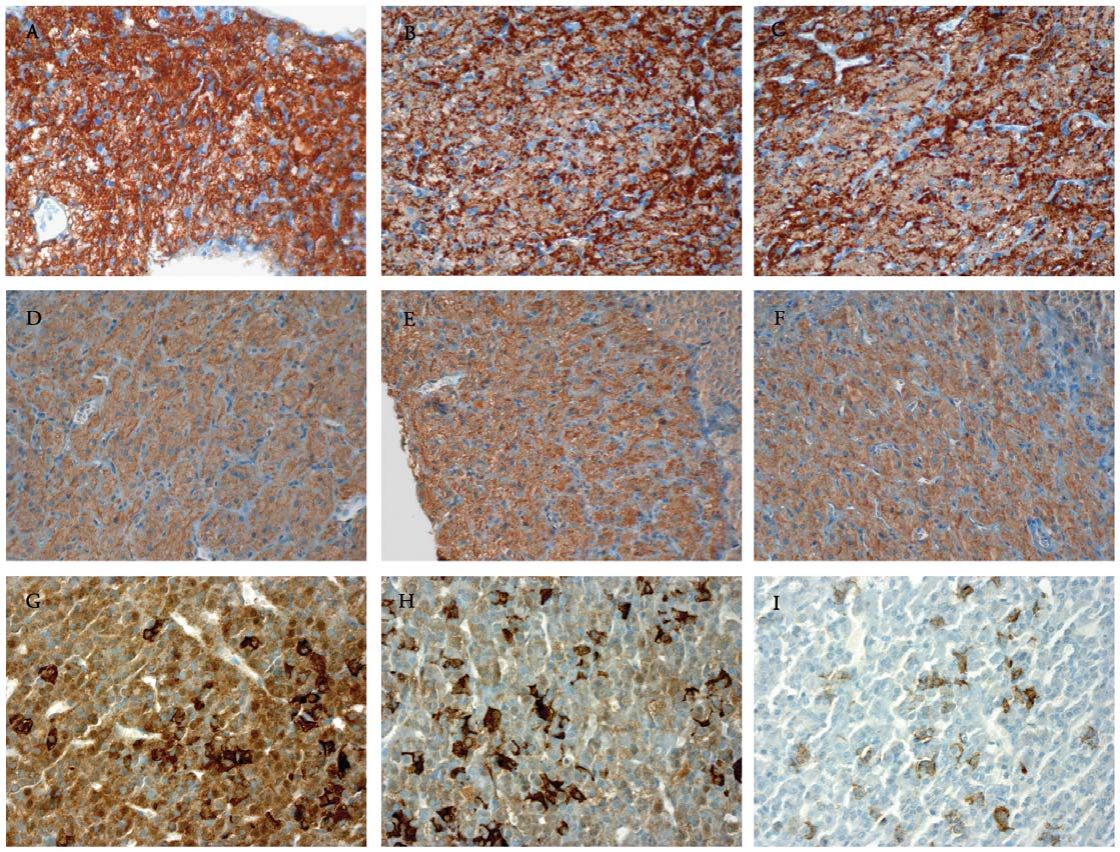
Ante-hypophysis of sham (A–D–G), septic (B–E–H) and septic with early death (septic ED) (C–F–I) rats. Labelling of CRHR1 (A–B–C) and V1b receptor (D–E–F) after immunohistochemistry (ABC peroxidase/DAB) did not vary among the three groups. ACTH (G–H–I) immunostaining (ABC peroxidase/DAB) was decreased in septic ED.

In patients, the antehypophyseal expression of ACTH was significantly lower in septic shock than in controls (*p* = 0.04) (Table 3 and Figure 3). Similarly, in rats, the antehypophyseal expression of ACTH varied significantly among groups (*p* = 0.002) (Table 3 and Figure 4). It was lower in septic shock than in either sham (*p* = 0.0004) or sepsis (*p* = 0.01) (Table 3 and Figure 3). It did not differ between sham and septic rats (*p* = 0.19) (Table 3 and Figure 4).

**Table 3:** Antehypophyseal expression of ACTH, CRHR1 and V1b receptor in control and septic shock patients and in sham, septic and septic ED rats.

	Humans			Rats			
	Controls	Septic Shock	p	Sham	Septic	Septic ED	p
CRHR1	3.0[2.3–3.0] Fig. 3A	2.0[2.0–3.0] Fig. 3B	0.66	3.0[2.0–3.0] Fig. 4A	2.5[1.2–3.0] Fig. 4B	2.0[2.0–2.0] Fig. 4C	0.71
V1b	2.0[1.3–2.8] Fig. 3C	1.8[1.1–2.0] Fig. 3D	0.44	2.0[2.0–2.0] Fig. 4D	2.0[2.0–2.0] Fig. 4E	2.0[2.0–2.0] Fig. 4F	0.44
ACTH	3.0[3.0–3.0] Fig. 3E	2.0[1.6–2.0] Fig. 3F	0.04	2.0[2.0–2.0] Fig. 4G	a2.0[1.5–2.0] Fig. 4H	b1.0[1.0–1.0] Fig. 4I	0.002

Abbreviations: ACTH, Adreno-Corticotropic Hormone; V1b, Vasopressin receptor 1b; CRHR1, Corticotropin Releasing Hormone receptor 1; ED, Early death.

Results are expressed as median (IQR). The index of labelling of ACTH, CRHR1 and V1b receptor immunohistochemistry ranged from 0 to 3.

Comparison between the following animal groups:

a Sham *versus* Sepsis,

b Sepsis *versus* Septic Shock,

c Sham *versus* Septic Shock.

In patients and in rats, expression of CRHR1 did not differ between groups (p = 0.66 and *p* = 0.71, respectively). It was the same for V1b receptor (p = 0.44 for both), as shown in table 3 and figures 3 and 4.

In ante-hypophysis expression of CRH R1, V1b and ACTH in patients did not correlate with duration of septic shock. The small number of patients treated with steroids preclude to assess their effect.

## Discussion

This study assessed sepsis-induced ACTH expression and expression of its main regulators, both in patients and in rats. Decreased ACTH and unchanged expression of AVP mRNA, CRHR1, and V1b receptors was a consistent finding in human and in rats. Conversely, CRH mRNA expression was increased in rats with septic shock but not in patients with septic shock. These results suggest that septic shock is associated with central impairment of the HPA, characterized by decreased ACTH synthesis while its two main regulators CRH and AVP remain roughly unchanged.

Exposure to an acute stressful stimulus is followed by rapid and self-limited increase in plasmatic ACTH and corticosterone [18], [19]. However, if the stimulus is repeated, plasma glucocorticoid levels are usually above basal values but plasma ACTH responses vary, being preserved or desensitized according to the nature of the stimulus [20], [21], [22], [23], [24]. Similarly to our study, in a cecal ligation and puncture (CLP) model, Carlson and colleagues showed an increase of CRH expression in PVN [25] and a reduced adrenocortical sensitivity to ACTH during acute and post-acute phase [26] as well as Oliveira-Pelegrin and colleagues showed no change in the paraventricular AVP expression between CLP and sham rats [27]. It has been shown that LPS down-regulates the mRNA coding for AVP and CRH antehypophyseal receptors; however,the effect of prolonged experimental sepsis on these receptors has not been assessed yet. Given that these conclusions are comparable with our findings, we think that the originality of our work lies on a broad description of the HPA both in human and animals, in post-acute and ultimate phase of septic shock. Additionally, one may argue that variation of variable value is low. This is due to the large interval of the scores that we used, which enabled to detect clear and obvious rather than subtle and disputable difference. It reflects also the reproducibility between evaluators and the standardization of brain sampling and experimental models.

Our results show that HPA axis appeared non-reactive. It seems unlikely that sepsis induced by the surgical model was not enough severe to activate HPA axis. Faecal peritonitis constitutes a sustained stimulus sufficient to induce tissue inflammation and impairment of visceral sensory pathways [28], [29], [30]. It has been previously showed that CLP reflects the clinical course of sepsis [9], [31] and all our septic animals showed features of severe illness. Moreover, this surgical model has been used to assess central neuroendocrine response [14], [25], which is likely to be present in septic patients.

Our study provides just a snapshot of the HPA axis's state at a given time of course of sepsis. Septic and septic shock animals were sacrificed or spontaneously died within 36 hours and patients died after several days of septic shock complicated by multiple organ failure. Thus, our description of HPA axis rather corresponds to the post-acute phase of sepsis in the experimental model, while that in septic patients to the ultimate phase. This difference may explain the finding that expression of CRH was greater in rats than in humans. Interestingly, a previous experimental study showed that parvocellular CRH expression peaked 24 hours after onset of CLP [25], suggesting that animals died in the post-acute phase of septic shock. To our knowledge there is no study that correlates neuropathological findings in HPA axis with the severity of the septic shock. It was not possible to determine whether the absence of increase in CRH depends on septic shock duration, intensity or both since all patients died from severe septic shock. The finding that CRH expression did not vary among patients according to the duration of septic shock (data not shown) supports that sepsis intensity was a determinant factor.

Another hypothesis to explain our results is based on the regulatory role of endogenous and exogenous glucocorticosteroids on HPA during stress. Indeed, cortisol exerts a negative feedback on PVN, reducing CRH and AVP mediated ACTH secretion [32].

Carlson and colleagues showed that plasma cortisol level decreased with time in CLP [26]. It is likely that similar kinetics took place in rats with faecal peritonitis, although we were not able to measure plasma cortisol level. Because of this decrease, it is unlikely then that endogenous glucocorticoids account for neuropathological findings in septic rats, notably decreased ACTH expression. None of the rats were treated with steroids. As plasma cortisol levels were not available in patients, we are not able to assess whether it correlated with CRH, AVP and ACTH secretion. One may argue that patient who had died from septic shock would have had increased plasma cortisol level. This is controversial. If increased plasma cortisol level at onset of septic shock, was associated with mortality [1], few studies have assessed whether this relationship remain along the course of septi shock. It has been reported that plasma cortisol level [33] or free cortisol level [34] decreased with time. It has been shown no difference in the decrease with time of free cortisol level [34] or in plasma cortisol levels before and during low–dose hydrocortisone therapy [35] between survivors between survivors and non survivors from multitrauma, sepsis [34] or septic shock [35]. We recently reported that plasma cortisol levels after 10 days in average from admission were higher in patients who will die than survive from severe critical illness [36]. It is therefore difficult to figure the relationships between plasma cortisol level and mortality along the course of the septic shock, the hypotheses that death is associated with high or low levels being both plausible,. Once again, small number of patients treated with steroids precludes to asssess their correlation with neuropathological findings.

The role of iNOS on neuroendocrine modulation of axis is well recognized and his expression may contribute to stunting of HPA in sepsis. Most experimental studies reported that nitric oxide stimulates the expression of CRH [37] and suppresses the stimulatory effect of AVP on ACTH secretion [38], [39]. It also plays an inhibitory action on AVP synthesis both in magnocellular [14] and parvocellular cells during sepsis [40]. The pro-apoptotic action of iNOS in PVN may also partially account for the reduced reactivity of HPA in sustained septic shock [41]. It would be interesting in a future experimental study to assess to what extent iNOS inhibition modifies the ante-hypophyseal system in septic and septic ED rats. We acknowledge that the present study is mainly descriptive and that we are not able to determine whether the observed phenomena were the cause of death. But we reasoned that before proposing experimental intervention (such as iNOS inhibition) a thorough description of ante-hypophyseal system was necessary.

In conclusion, the present study suggests that in humans and in rats, septic shock is associated with decreased ACTH synthesis that is not compensated by its two natural secretagogues AVP and CRH. One underlying mechanism might be increased expression of iNOS.

Further studies are necessary to assess other mechanisms and to elucidate the role of HPA impairment on relative adrenal failure in sepsis.

## References

[1] Annane D, Sebille V, Troche G, Raphael JC, Gajdos P (2000). A 3-level prognostic classification in septic shock based on cortisol levels and cortisol response to corticotropin.. JAMA.

[2] Annane D, Aegerter P, Jars-Guincestre MC, Guidet B (2003). Current epidemiology of septic shock: the CUB-Rea Network.. Am J Respir Crit Care Med.

[3] Martin GS, Mannino DM, Eaton S, Moss M (2003). The epidemiology of sepsis in the United States from 1979 through 2000.. N Engl J Med.

[4] Schroeder S, Wichers M, Klingmuller D, Hofer M, Lehmann LE (2001). The hypothalamic-pituitary-adrenal axis of patients with severe sepsis: altered response to corticotropin-releasing hormone.. Crit Care Med.

[5] Muller MB, Preil J, Renner U, Zimmermann S, Kresse AE (2001). Expression of CRHR1 and CRHR2 in mouse pituitary and adrenal gland: implications for HPA system regulation.. Endocrinology.

[6] Van Pett K, Viau V, Bittencourt JC, Chan RK, Li HY (2000). Distribution of mRNAs encoding CRF receptors in brain and pituitary of rat and mouse.. J Comp Neurol.

[7] Lolait SJ, Stewart LQ, Jessop DS, Young WS, O'Carroll AM (2007). The hypothalamic-pituitary-adrenal axis response to stress in mice lacking functional vasopressin V1b receptors.. Endocrinology.

[8] Hsieh CH, Li HY, Chen JC (2010). Nitric oxide and interleukin-1beta mediate noradrenergic induced corticotrophin-releasing hormone release in organotypic cultures of rat paraventricular nucleus.. Neuroscience.

[9] Wichterman KA, Baue AE, Chaudry IH (1980). Sepsis and septic shock–a review of laboratory models and a proposal.. J Surg Res.

[10] Hubbard WJ, Choudhry M, Schwacha MG, Kerby JD, Rue LW (2005). Cecal ligation and puncture.. Shock.

[11] Medina E (2010). Murine model of polymicrobial septic peritonitis using cecal ligation and puncture (CLP).. Methods Mol Biol.

[12] Heuer JG, Bailey DL, Sharma GR, Zhang T, Ding C (2004). Cecal ligation and puncture with total parenteral nutrition: a clinically relevant model of the metabolic, hormonal, and inflammatory dysfunction associated with critical illness.. J Surg Res.

[13] Pancoto JA, Correa PB, Oliveira-Pelegrin GR, Rocha MJ (2008). Autonomic dysfunction in experimental sepsis induced by cecal ligation and puncture.. Auton Neurosci.

[14] Sonneville R, Guidoux C, Barrett L, Viltart O, Mattot V (2009). Vasopressin Synthesis by the Magnocellular Neurons is Different in the Supraoptic Nucleus and in the Paraventricular Nucleus in Human and Experimental Septic Shock.. Brain Pathol.

[15] Sprung CL, Annane D, Keh D, Moreno R, Singer M (2008). Hydrocortisone therapy for patients with septic shock.. N Engl J Med.

[16] Paxinos G, Watson C (1997). The rat brain in stereotaxic coordinates.

[17] Polito A, Brouland JP, Porcher R, Sonneville R, Siami S (2011). Hyperglycaemia and apoptosis of microglial cells in human septic shock.. Crit Care.

[18] Herman JP, Figueiredo H, Mueller NK, Ulrich-Lai Y, Ostrander MM (2003). Central mechanisms of stress integration: hierarchical circuitry controlling hypothalamo-pituitary-adrenocortical responsiveness.. Front Neuroendocrinol.

[19] Lightman SL, Young WS (1988). Corticotrophin-releasing factor, vasopressin and pro-opiomelanocortin mRNA responses to stress and opiates in the rat.. J Physiol.

[20] Aguilera G (1994). Regulation of pituitary ACTH secretion during chronic stress.. Front Neuroendocrinol.

[21] Dohanics J, Linton EA, Lowry PJ, Makara GB (1990). Osmotic stimulation affects neurohypophysial corticotropin releasing factor-41 content: effect of dexamethasone.. Peptides.

[22] Holmes MC, Antoni FA, Aguilera G, Catt KJ (1986). Magnocellular axons in passage through the median eminence release vasopressin.. Nature.

[23] Irvine CH, Alexander SL, Donald RA (1989). Effect of an osmotic stimulus on the secretion of arginine vasopressin and adrenocorticotropin in the horse.. Endocrinology.

[24] Dallman MF, Akana SF, Cascio CS, Darlington DN, Jacobson L (1987). Regulation of ACTH secretion: variations on a theme of B.. Recent Prog Horm Res.

[25] Carlson DE, Chiu WC, Fiedler SM, Hoffman GE (2007). Central neural distribution of immunoreactive Fos and CRH in relation to plasma ACTH and corticosterone during sepsis in the rat.. Exp Neurol.

[26] Carlson DE, Chiu WC, Scalea TM (2006). Cecal ligation and puncture in rats interrupts the circadian rhythms of corticosterone and adrenocortical responsiveness to adrenocorticotrophic hormone.. Crit Care Med.

[27] Oliveira-Pelegrin GR, Ravanelli MI, Branco LG, Rocha MJ (2009). Thermoregulation and vasopressin secretion during polymicrobial sepsis.. Neuroimmunomodulation.

[28] Gaykema RP, Dijkstra I, Tilders FJ (1995). Subdiaphragmatic vagotomy suppresses endotoxin-induced activation of hypothalamic corticotropin-releasing hormone neurons and ACTH secretion.. Endocrinology.

[29] Kapcala LP, Chautard T, Eskay RL (1995). The protective role of the hypothalamic-pituitary-adrenal axis against lethality produced by immune, infectious, and inflammatory stress.. Ann N Y Acad Sci.

[30] Fleshner M, Goehler LE, Hermann J, Relton JK, Maier SF (1995). Interleukin-1 beta induced corticosterone elevation and hypothalamic NE depletion is vagally mediated.. Brain Res Bull.

[31] Mathiak G, Grass G, Herzmann T, Luebke T, Zetina CC (2000). Caspase-1-inhibitor ac-YVAD-cmk reduces LPS-lethality in rats without affecting haematology or cytokine responses.. Br J Pharmacol.

[32] Locatelli V, Bresciani E, Tamiazzo L, Torsello A (2010). Central nervous system-acting drugs influencing hypothalamic-pituitary-adrenal axis function.. Endocr Dev.

[33] Guzman JA, Guzman CB (2007). Adrenal exhaustion in septic patients with vasopressor dependency.. J Crit Care.

[34] Beishuizen A, Thijs LG, Vermes I (2001). Patterns of corticosteroid-binding globulin and the free cortisol index during septic shock and multitrauma.. Intensive Care Med.

[35] Oppert M, Reinicke A, Graf KJ, Barckow D, Frei U (2000). Plasma cortisol levels before and during “low-dose” hydrocortisone therapy and their relationship to hemodynamic improvement in patients with septic shock.. Intensive Care Med.

[36] Sharshar T, Bastuji-Garin S, De Jonghe B, Stevens RD, Polito A (2010). Hormonal status and ICU-acquired paresis in critically ill patients.. Intensive Care Med.

[37] Rivier C (2003). Role of nitric oxide in regulating the rat hypothalamic-pituitary-adrenal axis response to endotoxemia.. Ann N Y Acad Sci.

[38] Bugajski J, Gadek-Michalska A, Borycz J, Glod R (1999). Social stress inhibits the nitric oxide effect on the corticotropin-releasing hormone- but not vasopressin-induced pituitary-adrenocortical responsiveness.. Brain Res.

[39] Rivier C (1998). Role of nitric oxide and carbon monoxide in modulating the ACTH response to immune and nonimmune signals.. Neuroimmunomodulation.

[40] Oliveira-Pelegrin GR, de Azevedo SV, Yao ST, Murphy D, Rocha MJ (2010). Central NOS inhibition differentially affects vasopressin gene expression in hypothalamic nuclei in septic rats.. J Neuroimmunol.

[41] Sharshar T, Gray F, Lorin de la Grandmaison G, Hopkinson NS, Ross E (2003). Apoptosis of neurons in cardiovascular autonomic centres triggered by inducible nitric oxide synthase after death from septic shock.. Lancet.

